# Feasibility, safety and efficacy of endoscopic single-operator cholangioscopy: a retrospective single-center study

**DOI:** 10.1177/17562848241288111

**Published:** 2024-10-16

**Authors:** Karsten Büringer, Ulrike Schempf, Stefano Fusco, Dörte Wichmann, Dietmar Stüker, Martin Götz, Nisar P. Malek, Christoph R. Werner

**Affiliations:** Department of Gastroenterology, Hepatology, Gastrointestinal Oncology, Geriatrics and Infectious Diseases, University Hospital Tübingen, Otfried-Müller-Str. 10, Tübingen D-72076, Germany; Department of Gastroenterology, Hepatology, Gastrointestinal Oncology, Geriatrics and Infectious diseases, University Hospital Tübingen, Tubingen, Germany; Department of Gastroenterology, Hepatology, Gastrointestinal Oncology, Geriatrics and Infectious diseases, University Hospital Tübingen, Tubingen, Germany; Department of Gastroenterology, Hepatology, Gastrointestinal Oncology, Geriatrics and Infectious diseases, University Hospital Tübingen, Tubingen, Germany; Department of General, Visceral and Transplantation Surgery, University Hospital Tübingen, Tubingen, Germany; Department of Gastroenterology, Hepatology, Gastrointestinal Oncology, Geriatrics and Infectious diseases, University Hospital Tübingen, Tubingen, Germany; Medizinische Klinik IV, Gastroenterologie/Onkologie, Kliniken Böblingen, Böblingen, Germany; Department of Gastroenterology, Hepatology, Gastrointestinal Oncology, Geriatrics and Infectious diseases, University Hospital Tübingen, Tubingen, Germany; Department of Gastroenterology, Hepatology, Gastrointestinal Oncology, Geriatrics and Infectious diseases, University Hospital Tübingen, Tubingen, Germany

**Keywords:** retrospective observational study, single operator cholangioscopy

## Abstract

**Background:**

Endoscopic retrograde cholangiopancreaticography (ERCP) is the standard endoscopic procedure for the diagnosis and treatment of diseases of the pancreas and bile ducts. Cholangioscopy provides direct visualization of the bile ducts. It offers the possibility of more detailed diagnostic and therapeutic indications. Today, cholangioscopy is often performed as a single-operator (SOC) procedure.

**Objectives:**

We were interested in the clinical efficacy of our SOC procedure in comparison with published studies, and performed this retrospective data analysis of all our consecutive patients from 2016 to 2022 to analyze the feasibility, safety, and efficacy of SOC.

**Design and Methods:**

A retrospective single-center analysis of patients undergoing SOC at a tertiary center from 2016 to 2022 (*N* = 196) was performed. Demographic data, indication for SOC, exam-specific data, efficacy, and complications were included. Sensitivity and specificity for diagnosing indeterminate biliary strictures were calculated.

**Results:**

The most common indications for SOC were indeterminate biliary strictures (*n* = 117; 60%), treatment of biliary stones (*n* = 45; 23%), and other indications (*n* = 34; 17%), for example, foreign body removal or intraoperative SOC. In 97% of the SOC (*n* = 191), the procedure was technically successful. The diagnostic or therapeutic goal was achieved in 91% of SOC (*n* = 173). In the subgroup where the SOC result was confirmed by subsequent surgery (*n* = 93), sensitivity was 86%, specificity 99%, and SOC treatment of stones was successful in 89%. Complications occurred in (20%; *n* = 37). The majority of these patients (*n* = 18; 10%) had minor bleeding requiring no intervention.

**Conclusion:**

SOC is an effective and safe procedure that should be the standard of care when primary diagnostic and/or therapeutic ERCP has failed. The sensitivity and specificity for determining the dignity of biliary strictures and the efficacy for the treatment of difficult-to-treat stones are reproducibly very high.

## Plain language summary

Feasibility, safety and efficacy of endoscopic biliary endoscopy: a retrospective Single Center study

Imaging the bile duct with contrast medium and fluoroscopy as part of endoscopic retrograde cholangiopancreaticography (ERCP) is the gold standard. However, direct visualization of the bile duct using a small camera on a second endoscope (cholangioscope) opens up further diagnostic and therapeutic possibilities. This study is a retrospective study covering the years 2016-2022, with a total of 196 patients. Demographic data, indication for SOC, exam specific data, efficacy, complications were included. The most common indications for direct biliary endoscopy were indeterminate biliary strictures (n=117; 60%), treatment of biliary stones (n=45; 23%) and other indications (n=34; 17%), e.g. foreign body removal or intraoperative biliary endoscopy. In 97% of the direct biliary endoscopy (n=191), the procedure was technically successful. The diagnostic or therapeutic goal was achieved in 91% of direct biliary endoscopy (n=173). In summary, direct biliary endoscopy is an effective and safe procedure that should be the standard of care when primary diagnostic and/or therapeutic ERCP has failed.

## Introduction

Endoscopic retrograde cholangiopancreaticography (ERCP) is the standard procedure for the diagnosis and in particular, treatment of diseases of the pancreatic and biliary duct systems.

A disadvantage of ERCP is that only indirect image information is given due to changes, in contrast, filling of the ductal systems during fluoroscopy. However, direct visualization of the mucosal surface and the contents of the bile duct cannot be obtained. Indeed, ERCP is still hindered by low diagnostic sensitivity in defining the etiology of biliary strictures.^1^ In the literature, sensitivities for ERCP with brush cytology are given at 30%–72%, and for ERCP with biopsies at 43%–65%.^2–4^

Endoscopic sphincterotomy and stone extraction with ERCP are successful in more than 90% of cases. The stones are usually removed using balloon extraction catheters or wire baskets. Occasionally, large stones or impacted stones can be difficult to remove.^5^

In 10%–20% of cases with present biliary stones, conventional ERCP alone is not therapeutically sufficient, complementary techniques are required for complete stone removal.^6–8^

These diagnostic and therapeutic gaps are filled by cholangioscopy, which provides direct image information through a video chip at the distal end of the cholangioscope. Additionally, only cholangioscopy can be used to perform diagnostic and therapeutic procedures in the bile and pancreatic duct under direct visualization.^9^

There are different techniques of cholangioscopy: the “mother-and-baby” cholangioscopy, first described in 1970,^10^ with two operators for the respective mother-and-baby endoscope, and the so-called single-operator cholangioscopy, developed by Boston Scientific in 2007, where only one person operates both mother-and-baby endoscopes.^11^ In “direct” cholangioscopy, a small-caliber endoscope (albeit with a larger diameter than in “mother-and-baby” cholangioscopy) is perorally inserted directly into the bile duct. This has the advantage of a larger working channel but is generally much more technically demanding than a mother-and-baby cholangioscopy. In addition, direct cholangioscopes are rare and often only advanced prototypes.^12^ Today, cholangioscopy is mainly performed using the “mother-baby” technique. Cholangioscopes today are single-use disposable devices. The examiner inserts the cholangioscope over the working channel of the duodenoscope, which has already been placed in the duodenum and passed into the bile duct, usually guided by a wire.^13^ As only one examiner operates both the cholangioscope and the duodenoscope, the procedure is called single-operator cholangioscopy (SOC). Dual-operator cholangioscopy requires two examiners: one for the duodenoscope and one for the cholangioscope. This type of procedure is actually not very common.^14,15^

There are several single-operator cholangioscopes available on the market, very common are the Spyglass^®^ (Boston Scientific, Marlborough, MA, USA) and the EyeMax^®^ (MicroTech, Düsseldorf, Germany).

Cholangioscopy is indicated for the evaluation of indeterminate biliary strictures or for the endoluminal treatment of biliary stones that cannot be managed otherwise.^15–18^

Other indications include retrieval of migrated stents and other foreign bodies form the bile duct.^13^

Direct visualization makes it easier to differentiate between indeterminate biliary strictures in terms of benign and malignant lesions and to take targeted biopsies. In a prospective study, Kim et al.^19^ demonstrated that tumor vessels are highly specific cholangioscopic findings, indicating malignancy. They found irregularly dilated and tortuous vessels in 61% of patients with biliary malignancy and none in cases of benign biliary strictures.

Biliary and pancreatic stones were considered difficult-to-treat (1) if mobilization of the stone was unsuccessful during previous ERCP; (2) they had a larger diameter than 15 mm; (3) they were located in the intrahepatic or cystic duct, including Mirizzi syndrome; (4) they had a common bile duct (CBD) anomaly; or (5) the stones were impacted in the pancreatic duct.

If ERCP is not successful, extracorporal and intraductal lithotripsy remain as nonsurgical options. The SOC offers the option of intraductal, visually guided electrohydraulic lithotripsy (EHL), or laser lithotripsy (LL).^20^

Since we were interested in the clinical efficacy of our SOC procedure in comparison with published studies, we performed this retrospective data analysis of all our consecutive patients from 2016 to 2022 to analyze the feasibility, safety, and efficacy of SOC.

## Patients and methods

### Study

The reporting of this study conforms to the Strengthening the Reporting of Observational Studies in Epidemiology (STROBE) statement.^21^

We confirm that the results are reproducible with the help of the specified methods and patients.

### Ethics

The study was approved by the Ethics Committee of the Medical Faculty of the University of Tübingen (AZ: 304/2022BO2, date of approval 12 May 2022) and was conducted in accordance with the current version of the declaration of Helsinki.

### Patients

Demographic data, examination-specific data, and clinical data of patients who underwent SOC at the University Hospital of Tübingen between 2016 and 2022 were collected from the electronic patient records after querying the operation and procedure codes via internal controlling. The sample size was not calculated. The University Hospital Tübingen is a tertiary referral center localized in southwestern Germany (Region Neckar-Alb of the state Baden-Württemberg), which covers a population of 2.5 million inhabitants. The University Hospital Tübingen is a liver transplant center, a pediatric liver center, and a comprehensive cancer center. Thus, our endoscopy unit diagnoses and treats a wide spectrum of pancreatobiliary and hepatic diseases, including pediatric ERCP in children younger than 100 days. Between 2016 and 2022, *N* = 3708 ERCPS were performed at the University Hospital Tübingen with an average of 530 per year (interquartile range 502–557). After analyzing the database, the patients who had a SOC were selected, in sum *N* = 196 adult patients were included in this analysis.

### Inclusion/exclusion criteria

All patients over the age of 18 who received SOC at the University Hospital Tübingen were included. Patients under the age of 18 were excluded.

### Procedure details

A dedicated endoscopy assistant under the supervision of the endoscopist performed sedation according to German guidelines.^22^ Sedation was generally performed with propofol. In isolated cases, midazolam (*n* = 23) was administered additionally. General anesthesia with oropharyngeal intubation under the supervision of an anesthesiologist was performed in 11 patients. The SOC was always performed with fluoroscopy as part of the ERCP with the Pentax (Hoya Corporation, Tokyo, Japan) duodenoscope, in mother-baby technique using the Spyglass Cholangioscope (Boston Scientific). The SOC was performed by an experienced examiner (experienced after Onkozert guidelines, which means at least 50 ERCP/year). SOC was classified as *technically* successful if the insertion of the SOC into the bile duct was feasible. Between 2016 and 2022, *N* = 3708 ERCPs were performed at the University Hospital Tübingen. An average of *N* = 530 per year, (interquartile range 502–557). The procedure was considered *clinically* successful if the clinical treatment or the diagnostic goal could be achieved.

A single dose of antibiotics was administered before or during the examination in most patients.

In all examinations, the SpyGlass Cholangioscope (Boston Scientific) was used. The SpyBite^®^ forceps (Boston Scientific) were used for biopsy sampling in each case.

### Definitions

The SOC was classified as *technically* successful if the insertion of the SOC into the bile duct was feasible. The procedure was considered *clinically* successful if the clinical treatment or diagnostic goal could be achieved.

*Indeterminate biliary stricture* was defined as the narrowing of a focal section of the bile ducts on abdominal computed tomography (CT), magnetic resonance imaging (MRI), endoscopic ultrasound, or ERCP, which resulted in dilation of the bile duct above the stricture and no definite diagnosis of dignity could be made.^17^

In general, at least six biopsies were taken under direct visualization with SOC. All biopsies were taken with the SpyBite forceps and the samples were analyzed by the pathologist. As these are fine specimens, the sample was specially labeled. In the case of very fine specimens, the samples were centrifuged beforehand by the pathology department.

Inadequate samples (insufficient tissue removal for histological analysis) were classified as not clinically successful in the analysis. Valid biopsy samples were categorized as benign (nonmalignant) or malignant (adenocarcinoma, dysplasia, and atypical cells with suspected malignancy). Whether the diagnosis was correct, was calculated and validated by comparison with the subgroups of patients who underwent surgery, so a surgically obtained histological sample was available.

*Biliary and pancreatic stones* were considered difficult-to-treat, if mobilization of the stone was not successful in previous ERCP, larger than 15 mm, located in intrahepatic or cystic duct, in patients with Mirizzi’s syndrome or any CBD abnormality, impacted in pancreatic duct. In addition to EHL, mechanical lithotripsy, basket, and balloon traction were also used. An expansion papillotomy, bougienage, or dilatation often had to be performed first. The probes from Walz Elektronik GmbH, Rohrdorf, Germany were used for the EHL. With the EHL probe, three intensities can be used in a controlled manner, corresponding to a power of up to 950 mJ. In one case, the stone was fragmented using a laser probe (Boston Scientific; Lighttrail 270 µm).

*The adverse events* in this retrospective analysis, each complication (bleeding, cholangitis, pancreatitis, perforation, and cardiopulmonary) was analyzed, both in the previously performed ERCP with papillotomy and in the SOC. Bleeding was categorized into minor and major bleeding. Minor bleeding is defined as self-limited bleeding. Major bleeding means a laboratory hemoglobin drop, more than one point.

### Statistics

The data collected were documented in a database using Microsoft Excel (Microsoft Corporation, Redmond, WA, USA) and analyzed using GraphPad Prism (GraphPad Corporation, Boston, MA, USA) or Microsoft Excel, (Microsoft Corporation).

## Results

### Cohort characteristics

Between 2016 and 2022, 196 SOCs are performed. Baseline characteristics are listed in Table 1. Radiological imaging (CT and/or MRI) had been performed prior to the SOC in 85% (*n* = 166). At least one previous ERCP had been performed in 82% (*n* = 161) of patients. 79% (*n* = 154) had already received a sphincterotomy. Sedation during SOC was analyzed with a median propofol dose of 815 mg (interquartile range 450–1022.5/95% CI 677.7–810 mg).

**Table 1.** Demographics, values during examination, indications, and previous examinations of the cohort who had a SOC between 2016 and 2022.

Demographics	
Cholangioscopies 2016–2022	
Years	
2016–2022 Total	*N* = 196
Age	
Median	64.5 years
Interquartile range	52–76 years
95% CI	60.6–65 years
Gender	
Male	*n* = 116/59%
Female	*n* = 80/41%
Total	*N* = 196
Values during examination	
Cholangioscopies 2016–2022	
Dose of propofol during the whole examination (e.g., ERCP + SOC)	
Median	815 mg
Interquartile range	450–1022.5 mg
95% CI	677.7–810 mg
Indications	
Cholangioscopies 2016–2022	*n*/%
Indeterminate biliary stricture	117/60%
Endoluminal treatment of pancreaticobiliary stones	45/23%
Screening procedures (after liver transplantation, follow-up after treatment of carcinoma)	14/7%
Foreign body removal	7/4%
Intraoperative	6/3%
Screening by PSC	6/3%
Transhepatic rendezvous with transabdominal cholangiography (PTCD )	1/1%
Previous examinations	
Cholangioscopies 2016–2022	*n*/%
Previous imaging (CT or MRI)	
Yes	166/85%
No	30/15%
Total	196
Previous ERCP	
Yes	161/82%
No	35/18%
Total	196
Previous sphincterotomy	
Yes	154/79%
No	42/21%
Total	196

ERCP, endoscopy retrograde cholangiopancreticography; PSC, primary sclerosing cholangitis; PTCD, percutaneous transhepatical biliary drainage; SOC, single-operator cholangioscopy.

### Indications for SOC

**Figure 1.**
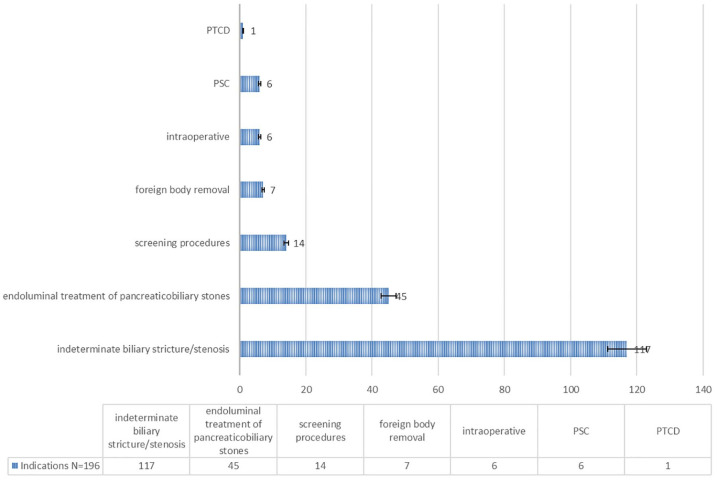
Distribution of indications for SOC in percent, the following are summarized under screening procedures: (SOC after liver transplantation, SOC for follow-up after treatment of carcinomas). SOC, single-operator cholangioscopy; PSC, primary sclerosing cholangitis; PTCD, percutaneous transhepatical biliary drainage.

**Figure 2.**
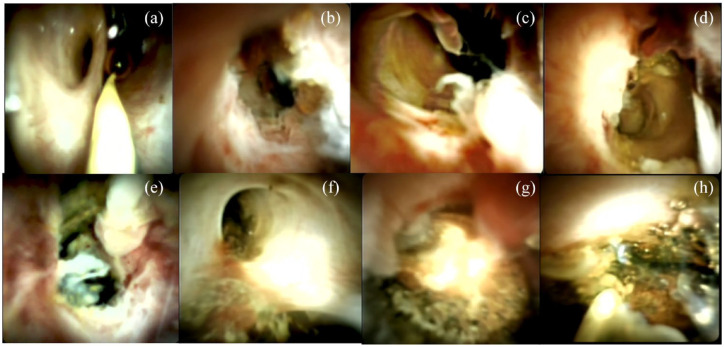
Most common indications for SOC. (a) Physiological CBD and orifice of the cystic duct with biopsy forceps. (b)–(d) Malignant strictures. (e) IgG4-related stricture. (f) Sludge in the bile duct. (g) Biliary stone. (h) Foreign body, impacted and torn off “Dormia” basket with biliary stone. SOC, single-operator cholangioscopy.

The most common indications for SOC were diagnosis of indeterminate biliary stricture in 60% (*n* = 117) and endoluminal treatment of pancreaticobiliary stones in 23% (*n* = 45, see Figures 1 and 2). Other indications were: screening procedures (after liver transplantation, follow-up after treatment of carcinomas; *n* = 14; 7%), foreign body removal (*n* = 7; 4%), intraoperative SOC (*n* = 6; 3%), screening for PSC (*n* = 6; 3%), and transhepatic rendezvous with transabdominal cholangiography (PTCD; *n* = 1; 1%; see Figure 1).

Indeterminate biliary stricture was defined as the narrowing of a focal section of the bile ducts on abdominal CT, MRI, endoscopic ultrasound, or ERCP, which resulted in dilation of the bile duct above the stricture and no definite diagnosis of dignity could be made.^17^ The second most common indication was the treatment of biliary-pancreatic stones. The average stone size was 16 mm (95% CI: 14, 7–17, 4 mm).

### Efficacy of SOC: Technical and clinical success

To assess the efficacy of SOC the technical and clinical success of the procedure was evaluated, see Table 2: SOC was technically successful in 97% (*n* = 191). Reasons for failure of intubation of the biliary or pancreatic duct was the technical failure of intubation of the papilla despite measures such as dilatation of our bouginage 3% (*n* = 5).

**Table 2.** Detailed information on the SOC performed between 2016 and 2022.

Cholangioscopies 2016–2022	*n*/%
Technical-successful	
Adjustable bile duct—cholangioscopy	
Yes	191/97%
No	5/3%
Total	196
Clinical-successful, in general	
Indication measure successful	
Yes	173/91%
No	18/9%
Total	191
Clinical-successful, subgroups	
Diagnostic indeterminate biliary stricture/stenoses	
Yes	108/95%
No	5/5%
Total	113
False negative histology (sensitivity)	
Yes	15/14%
No	93/86%
Total	108
“Difficult-to-treat” biliary stones	
Yes	54/96%
No	2/4%
Total	56^a^
Concrement size	
Mean, 95% CI	16 mm, 14.7–17.4 mm
Screening procedures (after liver transplantation, follow-up after treatment of carcinoma)	
Yes	13/93%
No	1/7%
Total	14
Foreign body removal	
Yes	6/85%
No	1/5%
Total	7
Intraoperative	
Yes	6/100%
No	0/0%
Total	6
Screening by PSC	
Yes	6/100%
No	0/0%
Total	6
Transhepatic rendezvous with transabdominal cholangiography (PTCD)	
Yes	0/0%
No	1/100%
Total	1
Adverse events	
Bleeding	
Yes major	9/5%
Yes minor	18/10%
Cholangitis/pancreatitis others (cardiopulmonary problem/perforation)	
Yes	6/2/4%
Yes	2/1%
Total	37
No adverse events	
No	154/80%
Total	191

ERCP, endoscopy retrograde cholangiopancreticography; PSC, primary sclerosing cholangitis; PTCD, percutaneous transhepatical biliary drainage; SOC, single-operator cholangioscopy.

a First indication other than an additional biliary stone.

SOC was clinically successful in 91% of technically successful SOCs (*n* = 173). For the most common indication, the clarification of indeterminate biliary stricture, we had a success rate of 95% (*n* = 108) for reaching the lesions and taking a biopsy.

In all cases, the SpyBite from Boston Scientific was used to take the biopsies. In each case, 6–10 biopsies were obtained. As the samples are very fine, they were specially labeled for the pathologists.

Removal of biliary and pancreatic stones by SOC, as the second most common indication (see above), was clinically successful in 96% (*n* = 54) of cases. In 89% (*n* = 50) of cases with biliary-pancreatic stones, an ERCP without complete removal of the stones had already been performed previously, often in another hospital. The stones were successfully removed in 54 (96%) cases, of which 51 were biliary stones and three were pancreatic stones. EHL was performed in 24 (44%) cases as part of the SOC; in one case, laser therapy with the Boston Scientific Light Trail was performed after an ineffective EHL. In addition to EHL, mechanical lithotripsy, basket- and balloon traction were used. An extension papillotomy, bougienage, or balloon dilatation often was performed first. In six cases (11%), more than one SOC sessions were required to extract the stones. In the other indications, for example, foreign body retrieval and intraoperative SOC, the success rate was 91% (*n* = 31).

### Indeterminate biliary stricture: Sensitivity and specificity of SOC

In the diagnosis of indeterminate biliary stricture, 96% (*n* = 108) of patients had the stenosis identified and biopsied by SOC. For the subgroup of patients who underwent subsequent surgery or had no evidence of malignancy in follow-up examinations (*n* = 93), the sensitivity and specificity of SOC could be calculated. In this group, out of 108 biopsies performed, 93 were true positives and 15 were false negatives. Thus, SOC had a sensitivity of 86% (*n* = 93) for biopsies. The calculation of specificity is 99% (*n* = 107) because there was one false positive biopsy.

In the patients with indeterminate biliary strictures, the most common condition was malignant stenosis (*n* = 83; 71%). The second most common group was patients with PSC (*n* = 25; 21%). Other diagnoses were in 8% (*n* = 9) of patients were biliary strictures after liver transplantation (*n* = 5), autoimmune hepatitis (*n* = 3), and IgG4-associated cholangitis (*n* = 1). In nine patients with an indication for clarification of indeterminate biliary strictures, biliary stones were also diagnosed as a secondary diagnosis.

Of the 15 cases that were false negative, 13 showed a CCC in the surgical obtained pathological specimen and one showed malignant cells of a CCC in the ascites. One false negative case showed serological evidence of alveolar echinococcosis. In the only one false positive case, there was in the surgical obtained pathological specimen evidence of IgG4-associated autoimmune cholangitis. With a calculated prevalence of 0.1, this results in a positive predictive value (PPV) of 0.905 and a negative predictive value (NPV) of 0.985. Without using the prevalence for the calculation, the likelihood ratio (LR) can be calculated using sensitivity and specificity. The calculated LR+ value is 86 and the LR− is 0.14, which shows that SOC has a high positive value as a diagnostic method for classifying indeterminate biliary strictures. The area under the curve of the ROC test is 0.869.

### Safety of SOC

In the cohort, SOC complications, see definitions, occurred in 20% (*n* = 37). The majority of adverse events were minor bleeding (10%, *n* = 17, non-relevant or self-limited bleeding) observed during the procedure without the need for intervention, mainly due to sphincterotomy during ERCP. Nine patients (5%) had major bleeding (hemoglobin reduction), all of which were managed conservatively with endoscopic hemostasis (*n* = 3 stent placement, *n* = 2 fibrin glue injection). Following SOC, cholangitis (*n* = 6) or pancreatitis (*n* = 2) occurred in 4% (*n* = 8) of the cohort. One patient experienced periinterventional tachyarrhythmia absoluta and respiratory failure during the study. One patient had a bile duct perforation during EHL. A stent was placed to bridge the perforation and the stone was removed surgically by laparotomy, open cholecystectomy and resection of the extrahepatic bile ducts. Seven days after the procedure, the patient was discharged from hospital.

## Discussion

This retrospective analysis was performed to evaluate the feasibility, safety, and efficacy of SOC at a tertiary hospital and center for biliopancreatic diseases (BPDs).

SOC has become a standard for direct visualization and minimally invasive endoluminal therapy of the biliopancreatic system.^23^ However, SOC is generally not a first-line diagnostic procedure, with 82% of patients having undergone ERCP prior to SOC. Imaging techniques such as MRCP and CT can exclude many differential diagnoses in patients with BPD. MRCP has a sensitivity for the detection of biliary strictures of 80%–85%.^24^ SOC is therefore used specifically for questions requiring histological confirmation or as a therapeutic adjunct. As a single-use device, the costs associated with the SOC should not be underestimated.^25^ In this analysis, the most common indications for SOC were unclear stenosis and stone treatment. Other indications were screening, foreign body removal, intraoperative SOC, and transhepatic transabdominal cholangiography (Figure 1). The frequency of these indications corresponds to published data already available.^26,27^

In terms of feasibility, SOC was defined as technically successful, if intubation of the biliary or pancreatic duct was given. This could be achieved in 97% of cases.

The most common reason for failure of intubation of the biliary or pancreatic duct were the technical failure of intubation of the papilla despite measures like dilatation or bouginage in 3% of cases. The impassable constriction stenoses or a non-intubable papilla with conventional intubation are also described in the literature.^18,28^ With respect to efficacy, the SOC was considered clinically successful, if with means of the procedure, the therapeutic goal could be achieved (e.g., clarification of the stenosis and removal of the stones). This was achieved in 91% of procedures, which is consistent with the literature.^9^ The 91% refer to the entire procedure ERCP with SOC.

Sensitivity and specificity for the subgroup of patients, in whom subsequent surgery allowed histopathological confirmation of the SOC biopsy result were calculated to analyze the efficacy of SOC. A sensitivity (Sn) of 86% and a specificity (Sp) of 99% were found for SOC in the evaluation of indeterminate biliary strictures. With a calculated prevalence of 0.1, this results in a PPV of 0.905 and a NPV of 0.985. The calculated LR+ value is 86, which shows that SOC has a high positive value as a diagnostic method for clarifying indeterminate biliary stricture in order to make the correct diagnosis. Other studies showed a sensitivity of 57.7%–100% and a specificity of 88.9%–100% for SOC in this indication.^9,14,15,20,27,29–31^ A meta-analysis by Siu and Tang^32^ found a pooled sensitivity of 60% and a specificity of 98% for SOC biopsy. Thus, the clinical efficacy in this study was in the same range as in previous studies, and overall SOC is an excellent tool for determining the dignity of biliary strictures.

Today, the diagnostic standard is transpapillary biopsy or brush cytology in cases of indeterminate stenosis. These two methods were compared in a systematic review and meta-analysis by Navaneethan et al.,^14^ which showed a pooled sensitivity of 45% for brush cytology and 48.1% for transpapillary biopsy. The specificity for both was 99%, with a sensitivity of 60%–86% and a specificity of almost 100%, depending on the study.^9,14,15,20,27,29–31^ SOC is the most sensitive method for further histological clarification of indeterminate biliary strictures/stenoses, underlining the clinical importance of this procedure, even though it is much more expensive than brush cytology or transpapillary biopsy.^1^

The treatment of “difficult-to-treat” biliary stones, see definitions, including large biliary stones, stone impaction in the biliary or cystic duct, intrahepatic location, stricture distal to stones, and anatomic variants causing challenging access to the biliary duct is another relevant indication for SOC.^33^ In the retrospective analyze, 54 cases of biliary-pancreatic stones were successfully removed, of which 51 were biliary- and 3 were pancreatic stones. The average stone size was 16 mm (95% CI: 14.7–17.4 mm). For these indication SOC was successful in this analysis in 96% of these cases. In a multicenter prospective study by Fugazza et al.,^15^ at 18 tertiary centers, the success rate for biliary stones 92.1% achieved by SOC. Other studies showed a clinical success rate of 86.1%–97.3% for biliary stones.^14,20,30,34–36^

In terms of safety, ERCP including SOC has the potential to cause bleeding, cholangitis, pancreatitis, and injury to adjacent structures.^15,33^ The joint examination of ERCP with SOC was found to be a safe procedure, with adverse events occurring in 20% of cases. Bleeding occurred in 15% and pancreatitis or cholangitis was observed in 4% of patients. In a study by Chandan et al. that published complications in the context of SOC, bleeding occurred in 15 cases out of 62 patients (24%).^37^ Subhash et al. reported a frequency of 4% for cholangitis and pancreatitis.^33^ A frequency of cholangitis or pancreatitis of 7% for SOC was found in the meta-analysis of 45 studies by Korrapati et al.^38^ In most studies, only the complications of SOC were described; in this analysis, we deliberately considered the entire procedure, as SOC without ERCP is not possible.

One limitation of this analysis is the retrospective design of the study and the small number of patients. The sample size was not calculated and may affect the statistical results. A prospective multicenter study is desirable. This would allow the sensitivity and specificity of the SOC to be better determined.

## Conclusion

In conclusion, the clinical implementation of SOC may be safe, feasible, and effective. The sensitivity and specificity for determining the dignity of biliary strictures and the efficacy for the treatment of difficult-to-treat stones are reproducibly very high. Actually, due to the high costs, SOC may be an important adjunct to conventional ERCP with fluoroscopy alone.

## Supplemental Material

sj-docx-1-tag-10.1177_17562848241288111 – Supplemental material for Feasibility, safety and efficacy of endoscopic single-operator cholangioscopy: a retrospective single-center study Supplemental material, sj-docx-1-tag-10.1177_17562848241288111 for Feasibility, safety and efficacy of endoscopic single-operator cholangioscopy: a retrospective single-center study by Karsten Büringer, Ulrike Schempf, Stefano Fusco, Dörte Wichmann, Dietmar Stüker, Martin Götz, Nisar P. Malek and Christoph R. Werner in Therapeutic Advances in Gastroenterology

## Appendix

### Abbreviations

BPD biliopancreatic disease

CBD common bile duct

CCC cholangiocellulary carcinoma

CI confidence intervall

CT computertomography

EHL electrohydraulic lithotripsy

ERCP endoscopic retrograde cholangiopan-creaticography

LL laser lithotripsy

MRI magnetic resonance imaging

OPS operation and procedure codes

PSC primary sclerosing cholangitis

PTCD percutaneous transhepatical biliary drainage

SOC single-operator cholangioscopy

## Declarations

**Ethics approval and consent to participate:** The study was approved by the Ethics Committee of the Medical Faculty of the University of Tübingen (AZ: 304/2022BO2, date of approval 12 May 2022) and was conducted in accordance with the current version of the declaration of Helsinki. Consent to participate was not necessary in the pseudonymized study setting.

**Consent for publication:** Informed consent was not necessary in the pseudonymized study setting.

**Availability of data and materials:** Data are available on request.
